# Supplementary material for: Enhancing biomedical relation extraction with directionality
Source: Bioinformatics. 2025 Jul 15;41(Suppl 1):i68–76. doi: 10.1093/bioinformatics/btaf226 (PMC12261447; doi:10.1093/bioinformatics/btaf226)
Supplement: btaf226_Supplementary_Data [file btaf226_supplementary_data.zip › btaf226_Supplementary_Data/Lai.91.article_al_text.docx]

**Figure 1.**

*Alt-text:*A screenshot of the text-mining-based annotation website, TeamTat, showing an example of an annotated PubMed abstract (PMID:9746003). The figure illustrates the annotation of a gene-gene relationship between the SAA gene and MAP kinases, with the directionality assigned (e.g., SAA as subject and MAP kinases as object). Entities and their roles are highlighted and labeled.

**Figure 2.**

*Alt-text:* A diagram of the proposed relation extraction model architecture. It shows the workflow starting from input document tokenization and chunking (prefix, infix, suffix), followed by soft and hard prompts integration, multi-task classification for relation, novelty, and directionality, and final output via max-pooling of chunk-specific predictions.

**Figure 3.**

*Alt-text:* An example of a model input prompt used for fine-tuning LLMs. It includes a prompt instructing the model to identify relation type, directionality, and novelty for a pair of named entities highlighted in the text and respond in a structured JSON format.

**Table 1.**

*Alt-text:* A table comparing dataset statistics between BioRED-BC8 and BC5CDR. It includes the number of training, development, and test abstracts, named entity pairs, relation types, total relation/novelty annotations, and directionality annotations.

**Table 2.**

*Alt-text:* A table showing F1-scores for various models, including GPT, LLaMA, and SOAT, on the BioRED test set, comparing performance across metrics involving entity pair, relation type, novelty, and directionality. The proposed model (ours) shows the best results in most categories, including multi-task training.

**Table 3.**

*Alt-text:* A table comparing precision, recall, F1-score, and standard deviation for different models on the BC5CDR test set. The proposed method shows the highest F1 score, slightly outperforming BioREx.

**Table 4.**

*Alt-text:* Ablation study results show the performance of models using different context chunks (prefix-only, infix-only, suffix-only, and full model). The complete model using all chunks performs best across most evaluation metrics.

**Table 5.**

*Alt-text:* Evaluation results of different soft prompt lengths on BioRED and BC5CDR datasets. Adding soft prompts achieves modest performance improvements, especially in entity pair classification.
