# Supplementary material for: Enhancing biomedical relation extraction with directionality
Source: Bioinformatics. 2025 Jul 15;41(Suppl 1):i68–76. doi: 10.1093/bioinformatics/btaf226 (PMC12261447; doi:10.1093/bioinformatics/btaf226)
Supplement: btaf226_Supplementary_Data [file btaf226_supplementary_data.zip › btaf226_Supplementary_Data/Lai.91.sup.1.pdf]

Annotation Guidelines for Directional Relations

The task is to identify the subject and object of two entities in each relation. The entities to be annotated are as follows:

- Gene (or protein)
- Chemical (or drug)
- Disease (or syndrome, symptom, phenotype)
- Genomic variant

BioRED Entity Pairs

The BioRED entity pairs are categorized as follows:

1. Gene-Gene
2. Chemical-Gene
3. Disease-Gene
4. Chemical-Disease
5. Chemical-Chemical
6. Variant-Chemical
7. Disease-Variant
8. Variant-Variant

Annotation Notes

- When annotating, it's crucial to capture the most meaningful and direct biological relationships. Each identified relationship should clearly denote the subject and object to maintain consistency and accuracy unless the context is ambiguous.

Here's a breakdown of the subjects, objects, and various cases for each type of entity pair.

Chemical (subject) – disease (object)

1. Treatment: A chemical/drug (Subject) treats or alleviates disease symptoms (Object), such as insulin is administered for diabetes.
2. Causality: A chemical/drug (Subject) causes the disease development (Object), for instance, prolonged exposure to carcinogens can lead to cancer.

Disease (subject) – chemical (object)

Certain diseases affect chemical levels in the body. Common cases include:

1. Endocrine diseases: For instance, diabetes (Subject) elevates glucose and affects insulin levels (Object).

2. Metabolic syndrome: Conditions like hyperlipidemia (Subject) may cause abnormal lipid levels (Object) in the bloodstream.
3. Liver or kidney diseases: These (Subject) impact chemicals/drugs (Object) metabolism.
4. Inflammatory diseases: Diseases (Subject) such as rheumatoid arthritis alter inflammatory markers (Object).
5. Cancer: Some cancers (Subject) lead to biochemical marker changes (Object).
6. Neurological Disorders: For instance, Alzheimer's (Subject) may affect neurotransmitter levels (Object).

<u>Variant (subject) – chemical (object)</u>

Genetic variants can impact how individuals respond to drugs, which is especially important in pharmacogenomics. Key types include:

1. Drug efficacy: Variants (Subject) enhance or diminish drug effectiveness (Object).
2. Drug metabolism: Variants (Subject) affect how quickly a drug (Object) is cleared from the body.
3. Drug sensitivity or Resistance: Some variants (Subject) increase or reduce sensitivity to specific drugs (Object).
4. Drug toxicity: Variants (Subject) increase the risk of adverse drug reactions (Object).

<u>Chemical (subject) – variant (object)</u>

A chemical can cause the genetic variants.

1. Mutagenesis: Certain chemicals (Subject) may cause mutations (Object) in DNA.
2. Epigenetic alterations: Chemicals (Subject) can induce changes in gene expression (Object) without altering the DNA sequence itself.
3. Indirect genetic modifications: Chemicals (Subject) might indirectly cause genetic variations (Object) through mechanisms like cellular stress.

<u>Chemical (subject) – gene (object)</u>

A chemical can impact the expression, function, or activity of a gene or protein in various ways:

1. Drug-target interaction: Chemicals (Subject) might target specific proteins (Object) to alter their activity.
2. Chemical regulation of gene expression: Chemicals (Subject) can modulate gene expression (Object), either stimulating or suppressing it.
3. Protein modification: Chemical (Subject) alterations such as phosphorylation or acetylation can change protein behavior (Object).

4. Signal transduction modulators: Chemicals (Subject) affect genes (Object) in cellular signaling pathways.
5. Enzyme Activity Modulation: Chemicals (Subject) affect enzyme (Object) activity, impacting biological processes.
6. Induction of protein degradation: Chemicals (Subject) induce protein (Object) degradation.
7. Chemical-Induced epigenetic changes: Chemicals (Subject) influence epigenetic markers that control gene (Object) expression. For instance, some dietary components can modify DNA methylation patterns, affecting gene expression.

<u>Gene (subject) – chemical (object)</u>

The gene or protein affects chemical activity, metabolism, or response. Here are some cases where the gene or protein acts as the subject:

1. Enzymatic breakdown of chemicals: Genes/enzymes (Subject) metabolize chemicals/drugs (Object). For instance, the CYP450 genes are responsible for drug metabolism in the liver.
2. Gene-controlled drug transporters: Genes/proteins (Subject) transport drugs (Object) across cell membranes. For example, ABC transporter genes influence chemotherapy drug movement in and out of cancer cells.
3. Gene-mediated drug resistance: Genes confer resistance to specific drugs. For instance, the MDR1 gene expression can lead to resistance to specific chemotherapy agents.
4. Gene-regulated chemical sensitivity: Genes determine cells' sensitivity to certain chemicals. For example, variations in the TP53 gene can affect cellular responses to DNA-damaging agents.
5. Receptor genes influence drug binding: Genes code for receptors to which drugs bind.
6. Genes affecting chemical signaling pathways: Genes are involved in signaling pathways targeted by specific drugs.

<u>Variant (subject) – variant (object)</u>

Genetic variations interact within an individual's genome. These interactions affect the overall phenotype, disease risk, or treatment response. Here are the cases:

1. Epistasis: The effect of one variant depends on another variant's presence or absence.
2. Modifier genes: Variants modify the effects of mutations in other genes.
3. Compound heterozygosity: Two different variants in the same gene, one inherited from each parent, together cause a disease or condition, whereas each variant alone would not cause the phenotype.

4. Synthetic lethality: Two variants alone are harmless, but their combination leads to cell death or severe dysfunction.
5. Polygenic interactions: Multiple genetic variants influence many traits and diseases. The combined effect of these variants can be additive or synergistic, leading to a more pronounced trait or higher disease risk.
6. Genetic buffering: One variant compensates for another, leading to a milder phenotype or reducing disease risk.
7. Pharmacogenomic interactions: Multiple genetic variants can influence an individual's response to drugs, and the interaction between these variants affects drug metabolism, efficacy, or the risk of side effects.

<u>Gene (subject) – disease (object)</u>

When discussing the relationship where a gene (subject) is related to a disease (object), there are several key types of interactions:

1. Causality: Mutations or alterations in a specific gene directly cause a disease. This is common in many genetic disorders. For example, mutations in the BRCA1 and BRCA2 genes are known to increase the risk of breast and ovarian cancer significantly.
2. Predisposition to disease: Certain genes can increase an individual's susceptibility to developing a disease. While these genes do not directly cause the disease, they can make an individual more prone to it when combined with environmental factors and lifestyle choices. For example, certain variants of the APOE gene increase the risk of developing Alzheimer's disease.
3. Disease progression and severity: Variations in specific genes can influence the progression and severity of a disease. For instance, in diseases like cystic fibrosis, different mutations in the CFTR gene can result in varying degrees of disease severity.
4. Genetic modifiers of disease: Some genes may not directly cause a disease but can modify its expression or the severity of its symptoms. These genes can influence how a disease presents and progresses in an individual.
5. Pharmacogenetics in disease: Certain genes can affect how a patient responds to treatments for a disease. For example, variations affect how a patient metabolizes medication, affecting the drug's efficacy and the risk of adverse effects.

<u>Disease (subject) – gene (object)</u>

In the relationship where a disease (subject) is related to a gene (object), the interactions typically focus on how the disease affects or is associated with the expression, function, or structure of specific genes. Here are some key types of these relationships:

1. Gene expression changes: Certain diseases can lead to changes in the expression levels of genes. For instance, in cancer, the disease process can upregulate (increase

the expression of) oncogenes or downregulate (decrease the expression of) tumor suppressor genes.

2. Genetic association: This refers to the identification of genes that are more frequently altered in a particular disease. While these genes might not directly cause the disease, their altered states are associated with the disease's presence or severity. For example, genes that show altered expression patterns in autoimmune diseases.
3. Secondary genetic modifications: Diseases, particularly those that are chronic or inflammatory in nature, can lead to secondary changes in the genome, such as DNA methylation or histone modification, which can alter gene expression.
4. Genetic susceptibility markers: Some diseases can help identify genetic markers that indicate susceptibility to other conditions. For instance, certain gene patterns identified in one disease might be indicative of a predisposition to another disease.
5. Impact on genetic function: Diseases can affect the function of specific genes, either through direct interaction with the gene product or through downstream effects in a biological pathway. For instance, a neurodegenerative disease might affect genes involved in neuron function and maintenance.

<u>Gene(subject) – gene (object)</u>

The interaction between two or more genes to control a single phenotype. Here are some key aspects:

1. Epistasis: The effect of one gene (Object) is modified by one or several other genes (Subject). For instance, the expression of a trait might depend on the presence or absence of certain alleles at another gene locus.

<u>Chemical (subject) – chemical (object)</u>

Different chemicals affect each other's behavior, properties, or activity. Here are some key types of chemical-chemical interactions:

1. Pharmaceutical interactions: When two or more drugs are taken together, they can interact in ways that enhance or diminish their effects or produce new effects. This includes synergistic effects (where the combined effect is greater than the sum of individual effects), antagonistic effects (where one chemical reduces the effect of another), and additive effects (where the combined effect is equal to the sum of individual effects).
2. Chemical reactions: Chemicals can undergo reactions with each other, forming new compounds with different properties. These reactions are fundamental to chemistry and can range from simple combinations to complex multi-step processes.
3. Solubility and precipitation: The presence of certain chemicals can affect the solubility of others, leading to precipitation or dissolution. This is important in areas like environmental chemistry and pharmaceutical formulation.

4. Catalysis: Some chemicals can catalyze reactions between other chemicals, either speeding up the reaction or allowing it to occur under different conditions. Catalysts are widely used in industrial processes.
5. Environmental interactions: In the environment, chemicals can interact in complex ways, affecting the behavior and fate of pollutants, nutrients, and other chemical species. These interactions can impact bioavailability, toxicity, and mobility of chemicals in the environment.
6. Binding and complex formation: Chemicals can interact through binding or complex formation, which can alter their properties and functions. This is important in biochemical processes and in the development of certain types of drugs, such as chelating agents.
7. pH and Ionic strength effects: The pH and ionic strength of a solution can influence chemical interactions, particularly in biological systems or industrial processes where the conditions need to be carefully controlled.
8. Thermodynamic interactions: These involve changes in energy and entropy that occur during chemical interactions, affecting the stability and equilibrium of the system.

Detailed Results

Table 1: Detailed performance of the BioREDirect system on the BC8-BioRED test set. *L2R* denotes the direction from the left-hand-side named entity (Subject) to the right-hand-side named entity (Object), and vice versa for *R2L*.

NE Pair (L2R/R2L/None)	Relation Type (RT)	Instances	RT F1-score	RT+Dir. F1-score
Chemical-chemical (has dir. 40%/ None 60%)	Association	98	25.48	8.92
	Comparison	13	14.29	14.29
	Conversion	13	0.0	0.0
	Cotreatment	168	55.78	54.18
	Positive_Correlation	58	65.28	55.56
	Negative_Correlation	60	52.46	48.09
Chemical-disease (89%,7%,4%)	Association	90	14.63	6.5
	Positive_Correlation	248	58.17	51.79
	Negative_Correlation	437	70.45	68.70
Chemical-gene (69%, 17%, 14%)	Association	408	52.15	25.24
	Bind	45	52.78	22.22
	Positive_Correlation	271	70.05	66.44
	Negative_Correlation	388	74.16	71.69
Chemical-variant (9%,82%,9%)	Association	70	21.58	15.83
	Bind	7	0.0	0.0
	Positive_Correlation	40	5.0	5.0
	Negative_Correlation	82	20.63	14.29
Disease-gene (1%, 91%,8%)	Association	1262	66.02	61.24
	Positive_Correlation	274	20.78	17.82
	Negative_Correlation	73	32.32	24.24
Disease-variant (93%, 0%, 7%)	Association	272	30.69	27.39
	Positive_Correlation	664	56.22	55.92
	Negative_Correlation	39	57.14	54.76
Gene-gene (has dir. 62%/ None 38%)	Association	556	46.59	19.49
	Bind	79	46.99	31.32
	Positive_Correlation	193	49.51	46.58
	Negative_Correlation	108	44.61	43.12
